# Unraveling Eumelanin Radical Formation by Nanodiamond Optical Relaxometry in a Living Cell

**DOI:** 10.1021/jacs.3c07720

**Published:** 2024-03-12

**Authors:** Qi Lu, Berlind Vosberg, Zhenyu Wang, Priyadharshini Balasubramanian, Maabur Sow, Carla Volkert, Raul Gonzalez Brouwer, Ingo Lieberwirth, Robert Graf, Fedor Jelezko, Martin B. Plenio, Yingke Wu, Tanja Weil

**Affiliations:** † Max Planck Institute for Polymer Research, Ackermannweg 10, 55128 Mainz, Germany; ‡ Institute of Theoretical Physics and Center for Integrated Quantum Science and Technology (IQST), Ulm University, Albert-Einstein-Allee 11, 89081 Ulm, Germany; § Key Laboratory of Atomic and Subatomic Structure and Quantum Control (Ministry of Education), and School of Physics, South China Normal University, Guangzhou 510006, China; ∥ Guangdong Provincial Key Laboratory of Quantum Engineering and Quantum Materials, and Guangdong-Hong Kong Joint Laboratory of Quantum Matter, South China Normal University, Guangzhou 510006, China; ⊥ Institute for Quantum Optics and Center for Integrated Quantum Science and Technology (IQST), Ulm University, Albert-Einstein-Allee 11, 89081 Ulm, Germany

## Abstract

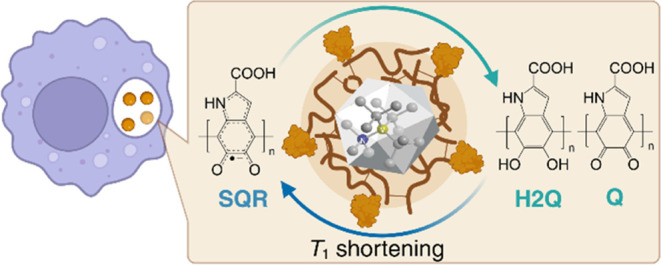

Defect centers in a nanodiamond (ND) allow the detection of tiny magnetic fields in their direct surroundings, rendering them as an emerging tool for nanoscale sensing applications. Eumelanin, an abundant pigment, plays an important role in biology and material science. Here, for the first time, we evaluate the comproportionation reaction in eumelanin by detecting and quantifying semiquinone radicals through the nitrogen-vacancy color center. A thin layer of eumelanin is polymerized on the surface of nanodiamonds (NDs), and depending on the environmental conditions, such as the local pH value, near-infrared, and ultraviolet light irradiation, the radicals form and react in situ. By combining experiments and theoretical simulations, we quantify the local number and kinetics of free radicals in the eumelanin layer. Next, the ND sensor enters the cells via endosomal vesicles. We quantify the number of radicals formed within the eumelanin layer in these acidic compartments by applying optical relaxometry measurements. In the future, we believe that the ND quantum sensor could provide valuable insights into the chemistry of eumelanin, which could contribute to the understanding and treatment of eumelanin- and melanin-related diseases.

## Introduction

Melanin is the primary photoprotecting pigment in humans and plays a crucial role in shielding our eyes and skin from the harmful effects of sunlight. However, its intricate involvement in various processes extends beyond photoprotection, including pigmentation regulation, efficient free radical scavenging, and even defense against intense radiation.^1−3^ Scientists are keen to elucidate the chemistry and biology of these pigments within living systems. The structure of the abundant black-brown polymeric pigment eumelanin is very heterogeneous and complex. It contains multiple indole building blocks, i.e., 5,6-dihydroxyindole and 5,6-dihydroxyindole-2-carboxylic acid, that are covalently and noncovalently linked, providing a variety of redox-active quinone, hydroquinone, quinone methide, and quinone imine groups.^4^ The generation of persistent, stable free radicals is a key feature of eumelanin physiochemistry.^5,6^ Many studies have been carried out using electron paramagnetic resonance (EPR) spectroscopy to investigate the spin properties of eumelanin in diverse conditions,^7−11^ and also using infrared spectroscopy to study the comproportionation reaction of eumelanin.^12^ The investigation of the formation of eumelanin radicals in the complex, dynamic, and inhomogeneous environment of living cells has not yet been achieved due to the lack of sensitivity of conventional detection methods. Moreover, the number of free radicals of eumelanin granules in different intracellular locations and at different time points can vary substantially.^13,14^ To gain a more comprehensive understanding of their biological functions in a spatiotemporal context, there is an urgent need for nanoscale characterization tools that allow for in situ real-time detection of the radicals present in eumelanin granules, with a particular emphasis on accurately quantifying the number of radicals formed inside cells.

Fluorescent nanodiamonds (NDs) with nitrogen-vacancy (NV^–^) centers can sense paramagnetic species such as ferritin,^15^ Gd^3+^,^16^ and radicals,^17−19^ due to their unique spin-dependent emission features. Furthermore, compared to conventional fluorescence-based methods of detecting radicals within living cells,^20−22^ NDs are inert, and they do not undergo chemical reactions with locally formed radicals. Since they neither interfere nor react with radicals formed during the detection process,^23^ they can outperform fluorescent dyes as probes that react with locally formed radicals^20−22^ to correlate radical formation with cellular responses.

**Figure 1:**
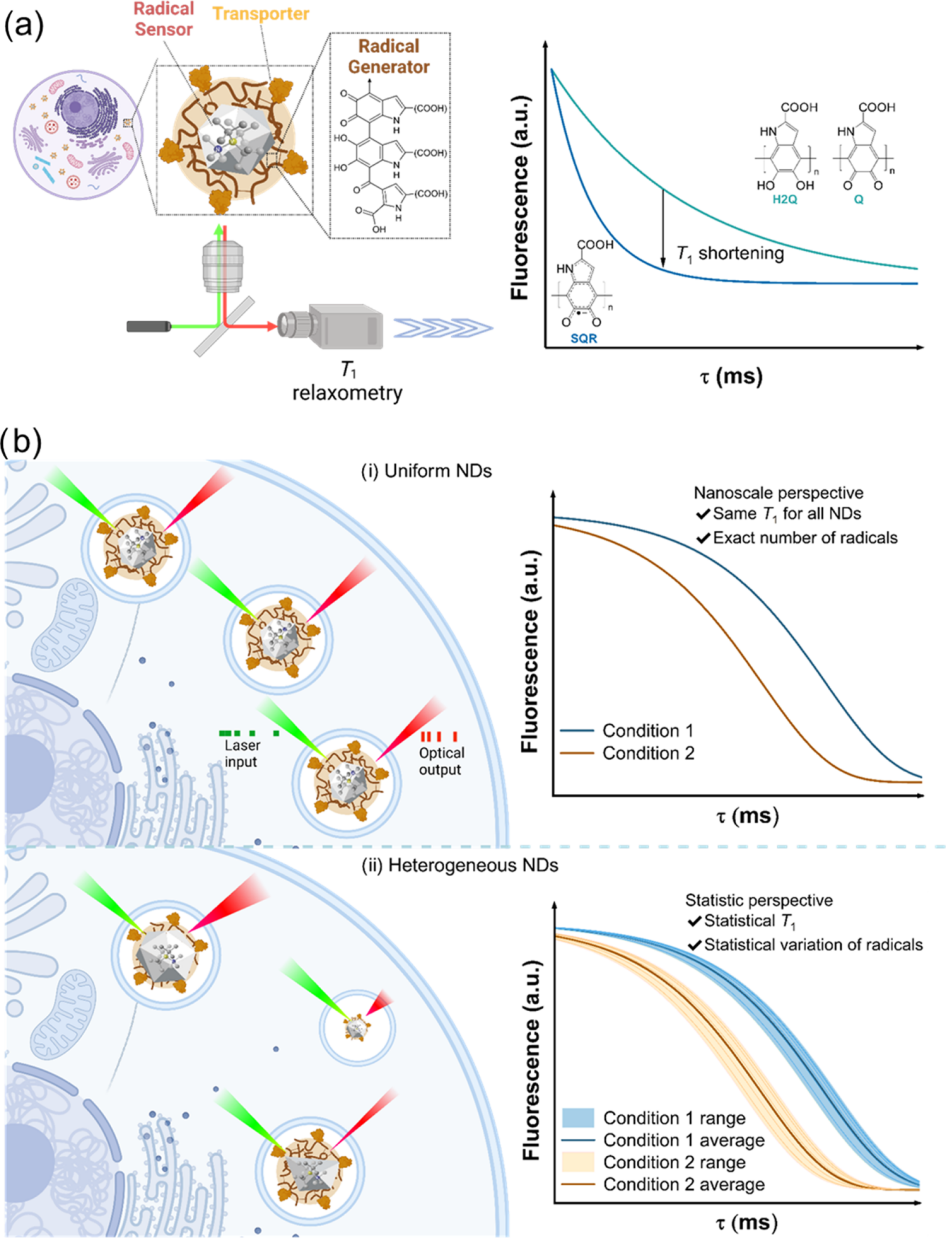
(a) Detecting free radicals of eumelanin in different media including the living cell. The ND sensor with an eumelanin layer allows radical generation and sensing by a fluorescence readout. A transporter protein is also applied to facilitate studies inside living cells. The transformation of eumelanin containing quinone (Q) and hydroquinone groups (H2Q) to semiquinone radicals (SQR) results in a shortening in *T*_1_ relaxometry, which allows in situ quantification of radicals in various media including complex living environments. (b) Illustration of quantification at the nanoscale and from a statistical point of view. (i) If all NDs have uniform characteristics, including size, shape, location of NV centers, presence of noisy electron spins, and composition of the eumelanin layer, the *T*_1_ time remains the same for all NDs under all pH conditions. (ii) Under experimental conditions, the NDs are heterogeneous; thus, multiple measurements have to be performed and statistical deviations occur.

The NV^–^-based quantum sensing methodology is depicted in Figure 1a. The *T*_1_ relaxation time of the NV^–^ centers in the ND sensor is determined by first initializing the NV^–^ in the *m*_s_ = 0 state using a green laser pulse. Following a variable waiting time, τ, the NV^–^ spin state is read out using a subsequent laser pulse. The *T*_1_ relaxation time is measured using this all-optical relaxometry technique. When NDs are exposed to a fluctuating magnetic field produced by the surrounding radicals, the *T*_1_ relaxation time of the NV^–^ centers is shortened. Consequently, a quantitative determination of the number of radicals surrounding the ND surface can be achieved with high sensitivity and high spatial resolution.^24^

In this study, we present an ND quantum sensor, termed radical generation and sensing nanodiamond (RGS-ND), that allows, for the first time, the sensitive in situ detection of radical species formed in eumelanin in a spatiotemporal manner after irradiation, or at different pH levels, and even inside a living cell. Following a previously published protocol,^25^l-DOPA has been polymerized on the surface of NDs into a thin and highly cross-linked network, a functional mimic of natural eumelanin.^26,27^ The absolute number of radicals detected by the RGS-ND quantum sensors has been quantified at different pH levels, and the kinetics of radical formation has been monitored in situ by theoretical and numerical modeling of the magnetic noise induced by the radicals in the eumelanin network. Subsequently, the method has been applied and tested in living cells, and in this way, the in situ detection of eumelanin radicals in acidic endosomal compartments has been realized. We believe that our method will contribute to the fundamental understanding of eumelanin chemistry and melanin-related cellular activities and diseases such as melanogenesis and melanoma.^28,29^

## Results and Discussion

### Preparation and Characterization of the Nanodiamond Radical Sensor

Fluorescent nanodiamonds (NDs) ranging from 40 to 50 nm dimensions were purchased from Adámas Nanotechnologies and used without further surface treatment. These comparatively smaller NDs were applied because they provide NVs closer to the surface compared to larger (>100 nm) NDs so that the fluctuations of the magnetic field have a stronger effect on *T*_1_. In addition to the size, also the ND morphology is an important parameter that affects spin sensitivity of the NDs.^30^ Therefore, transmission electron microscopy (TEM) and atomic force microscopy (AFM) were performed to measure the size and thickness of the NDs. An average diameter of 27.9 ± 13.1 nm (*n* = 50) and an average thickness of 5.65 ± 1.47 nm (*n* = 25) were determined (see Figure S1), indicating that the NDs have disk-like geometry.

**Figure 2:**
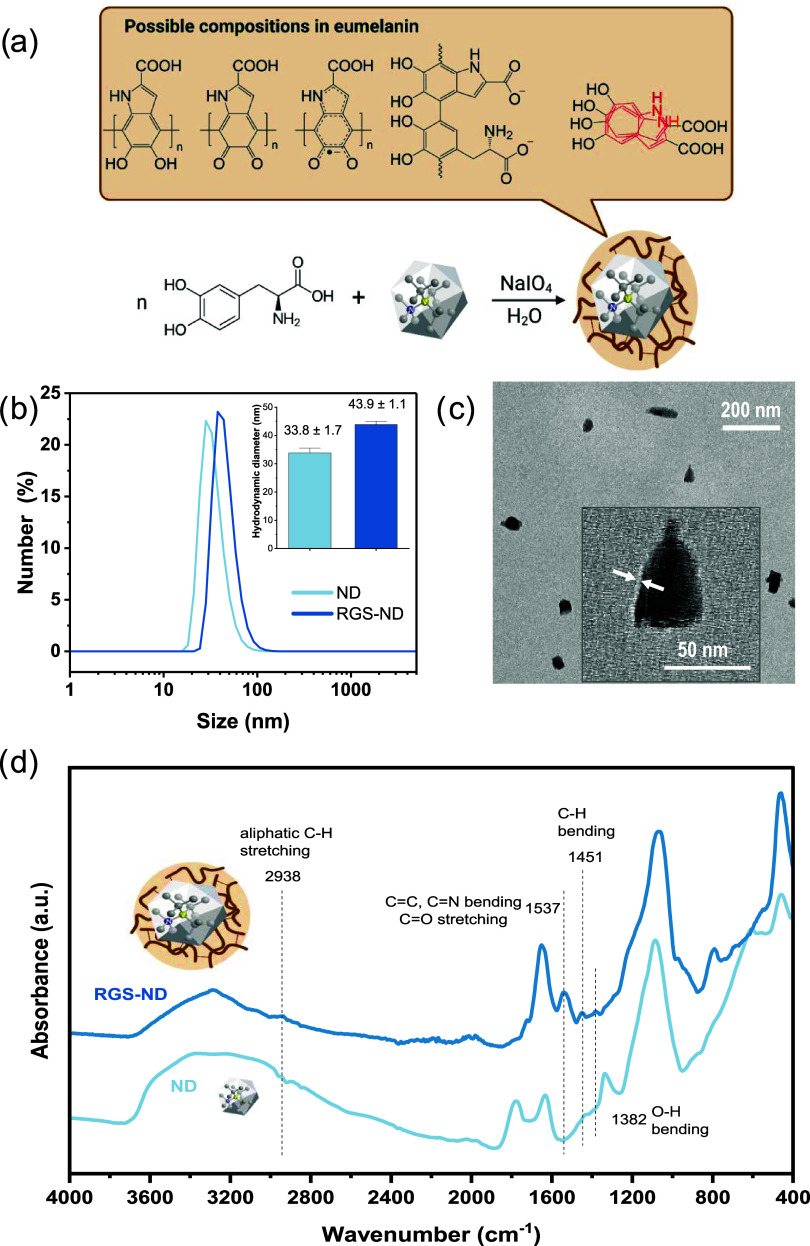
Characterization of RGS-NDs with a eumelanin surface layer. (a) Synthesis of RGS-NDs. (b) Hydrodynamic diameters determined by DLS (data presented as mean ± standard deviation (SD) of three measurements). (c) TEM image of RGS-NDs, the eumelanin layer was highlighted by the arrows. (d) Attenuated total reflectance-Fourier transform infrared spectroscopy (ATR-FTIR). 2938 cm^–1^: aliphatic C–H stretching; 1537 cm^–1^: aromatic ring C=C and C=N bending in addition to C=O stretching of carboxylic groups; 1451 cm^–1^: aliphatic C–H bending; 1382 cm^–1^: phenolic OH bending.

The eumelanin layer was then introduced onto the surfaces of the NDs. NDs were mixed with l-DOPA in an aqueous solution before the NaIO_4_ solution was added to initiate the oxidation of l-DOPA (Figure 2a). After stirring for 15 min, the unreacted reagents were removed by centrifugation at 12,000 rpm and washed three times with water to isolate the purified RGS-NDs in 23% yield. The hydrodynamic diameter of the RGS-NDs was assessed by dynamic light scattering (DLS) and reported as mean number distribution (Figure 2b). An increase from 33.8 ± 1.7 nm for the uncoated NDs to 43.9 ± 1.1 nm for the RGS-NDs was observed in aqueous media, corresponding to a thickness of the hydrated eumelanin layer of about 5 nm.

The coated RGS-NDs were further characterized by TEM to image their structure and morphology in a dry state. As shown in Figure 2c, TEM images revealed a uniform thin coating of about 2.0 ± 0.3 nm (*n* = 15) thickness surrounding the RGS-NDs in the dry state, indicating the presence of the eumelanin polymer. To further confirm the eumelanin layer on the surface of the NDs, the attenuated total reflection-Fourier transform infrared (ATR-FTIR) spectra of RGS-NDs were measured (Figure 2d). The weak intensity peak at 2938 cm^–1^ was attributed to the stretching vibration of the aliphatic C–H group.^31,32^ The peak at 1537 cm^–1^ was assigned to the bending vibration of aromatic ring C=C and C=N bonds of the aromatic system in addition to C=O double bonds of the carboxylic groups.^33^ The aliphatic C–H groups were found at 1451 cm^–1^. The weak signal at 1382 cm^–1^ could be assigned to the OH bending of phenolic groups, indicating the indole ring vibration.^31^ In addition, the absorption spectrum (Figure S2) of RGS-NDs showed a red shift of 5 nm compared to pure NDs due to the presence of the phenyl groups in the eumelanin layer.

### Nanoscale Detection of Radicals Formed in Eumelanin by Nanodiamond Optical Relaxometry

**Figure 3:**
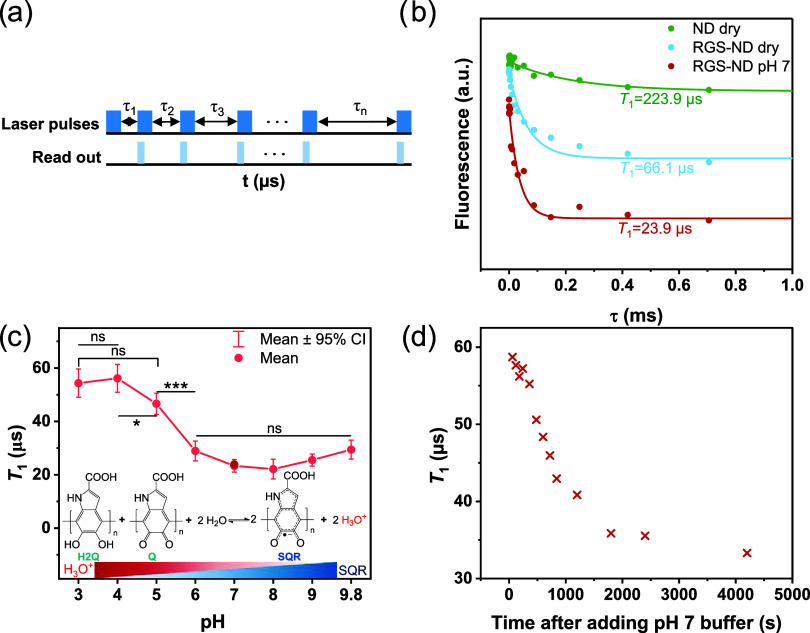
(a) Pulse scheme for *T*_1_ measurements. (b) Typical *T*_1_ measurement of NDs (green), RGS-NDs (blue) in dry conditions, and RGS-NDs in phosphate buffer at pH 7 (red). (c) *T*_1_ of RGS-NDs in buffer solutions (*n* ≥ 16). The comproportionation equilibrium of quinone (Q) and hydroquinone (H2Q) groups to form semiquinone radicals (SQR) is illustrated. (d) Kinetics of radical formation quantified by RGS-NDs over time after addition of phosphate buffer (pH 7) to the dry RGS-NDs. The significance level was 0.05 for mean comparison, *p < 0.05, *** *p* < 0.001, ns = not significant.

At the comproportionation equilibrium of eumelanin, the quinone (Q) and the hydroquinone groups (H2Q) in the eumelanin layer can react with water molecules to form hydronium ions and semiquinone radicals (SQR) as depicted in Figure 3c. In brief, the H2Q is first deprotonated to form Q^2–^, followed by one-electron oxidation to afford the radical SQR^34^ that can be detected on the surface of single NDs. To quantify the SQR radicals in the eumelanin layer of RGS-NDs, the longitudinal *T*_1_ relaxation time of the NV^–^ center was measured on a home-built optically detected magnetic resonance (ODMR) spectroscope. In brief, the radicals in the eumelanin layer generate a fluctuating magnetic field due to noise in the vicinity of the RGS-NDs that shortens the *T*_1_ relaxation time of the NV^–^ center, which is measured by optical readout.^35^ To perform these measurements, a silicone gasket was placed on top of an O_2_-plasma-cleaned glass coverslip, and the NDs and RGS-NDs dispersed in aqueous solution were drop-casted into the well and covered with a removable transparent plastic film to prevent evaporation of the buffer solution. First, the *T*_1_ relaxation time was determined on single, isolated RGS-NDs based on the confocal images depicted in Figure S3 at different pH levels. The pulse scheme for measuring the *T*_1_ relaxation time of the NVs is shown in Figure 3a. Subsequently, the *T*_1_ relaxation time was determined by first initializing the NV^–^ into the *m*_s_ = 0 state with a green laser pulse. After a variable waiting time, τ, the NV^–^ spin state was read out using a laser pulse to probe spin relaxation from the *m*_s_ = 0 spin state to the thermally mixed state. In Figure 3b, we plot the typical *T*_1_ measurements of dry ND (green), dry RGS-ND (blue), and RGS-ND dispersed in phosphate buffer at pH 7 (red). For each reaction condition (dry and buffer), the spectral properties of around 20 NDs were measured. We observed that the *T*_1_ relaxation time decreased from 223.9 μs for ND to 66.1 μs for RGS-ND (dry conditions) and to 23.9 μs for RGS-ND (phosphate buffer, pH 7). These observations are consistent with published work by Meredith et al.,^8^ in which they investigated changes in the radical concentration of an eumelanin film by EPR, indicating that water molecules support radical formation in the eumelanin layer.

Next, the impact of the pH value on radical formation and the mean *T*_1_ relaxation time in the eumelanin layer of RGS-NDs was evaluated. As shown in Figure 3c, the mean *T*_1_ relaxation times of RGS-NDs remained constant (54.4 ± 11.3, 56.2 ± 11.2 μs, *n* ≥ 16) at pH 3 and pH 4. This observation is in agreement with EPR data from the literature,^36^ indicating that the SQR forms the respective Q and H2Q species below pH 5. However, between pH 5 and 7, the mean *T*_1_ relaxation time of RGS-NDs gradually decreased from 46.6 ± 8.5 μs (pH 5) and 28.9 ± 8.1 μs (pH 6) to 23.3 ± 5.0 μs (pH 7), reflecting the shift in the comproportionation equilibrium toward the formation of SQRs, which reached saturation at pH 8. Further increases of the pH level up to 9.8 did not change the number of SQRs, and the mean *T*_1_ relaxation time remained constant. Above pH 10, eumelanin is known to degrade,^37^ and therefore, no measurements were performed at a higher pH level.

To investigate the kinetics of radical formation at pH 7, we conducted *T*_1_ relaxation time measurements of RGS-NDs immediately after adding the pH 7 buffer solution and monitored the changes in *T*_1_ over the course of approximately 1 h. Within the first 20 min after adding the buffer solution, we observed a significant decrease in *T*_1_, which then stabilized at around 35 μs (Figure 3d). This decrease was attributed to the formation of radicals within the eumelanin layer, which occurred immediately after the reaction was initiated by adding the buffer solution and remained constant after 20 min. These results could indicate that it takes about 20 min for a stable comproportionation equilibrium to form within the eumelanin shell. Possibly, the highly cross-linked eumelanin layer has an impact on the diffusion of water molecules required for the formation of hydronium ions and SQR at neutral pH. For comparison, we also recorded the continuous-wave (cw) EPR spectrum of RGS-NDs in different pH buffer solutions (Figure S4), and the measurements show the same trend of radical density as the *T*_1_ measurements (Figure 3c). The ratio between low and high pH samples, however, is significantly larger in the cw-EPR measurements. This is because the radicals in the RGS-ND samples are not distributed homogeneously in solution, but they are densely located only within the eumelanin layer, which are not fully saturated in our cw-EPR measurements. Although both cw-EPR and the *T*_1_ time measurements show the same trend, the EPR results do not provide quantitative information on the number of radicals in the eumelanin layer, thus highlighting the considerably higher sensitivity of *T*_1_ relaxometry compared to bulk EPR measurements.^38^

### Nanoscale Detection of Local Temperature Changes by Nanodiamond Optical Relaxometry

Melanin can reduce the risk of skin cancer by transforming the absorbed sunlight into heat energy.^39,40^ Therefore, the influence of local temperature fluctuations on the RGS-NDs was investigated first by measuring the *T*_1_ relaxation time under NIR irradiation (810 nm, 350 mW/cm^2^) for 10 min because the *T*_1_ relaxation time only shows temperature-dependency at cryogenic temperatures. Therefore, only the formation of SQ radicals within the eumelanin shell should shorten the *T*_1_ relaxation time in our setup.^41^ RGS-NDs dispersed in water and in the dry state were irradiated for 10 min, and the *T*_1_ relaxation times remained constant as depicted in Figure S5. Next, the mean *T*_1_ relaxation times of RGS-NDs were measured under UV irradiation (365 nm, 0.3 mW/cm^2^) for 10 min, and the corresponding results were presented in Figure S6. No significant changes were observed with or without irradiation with UV or NIR light, indicating that RGS-NDs selectively respond to changes in local radical formation, while UV radiation and potential local temperature gradients^42^ did not interfere with radical sensing in our experimental setup.

### Quantification of Radical Formation in the Eumelanin Shell of RGS-NDs by Theoretical Simulation of Spin Relaxation Times

**Figure 4:**
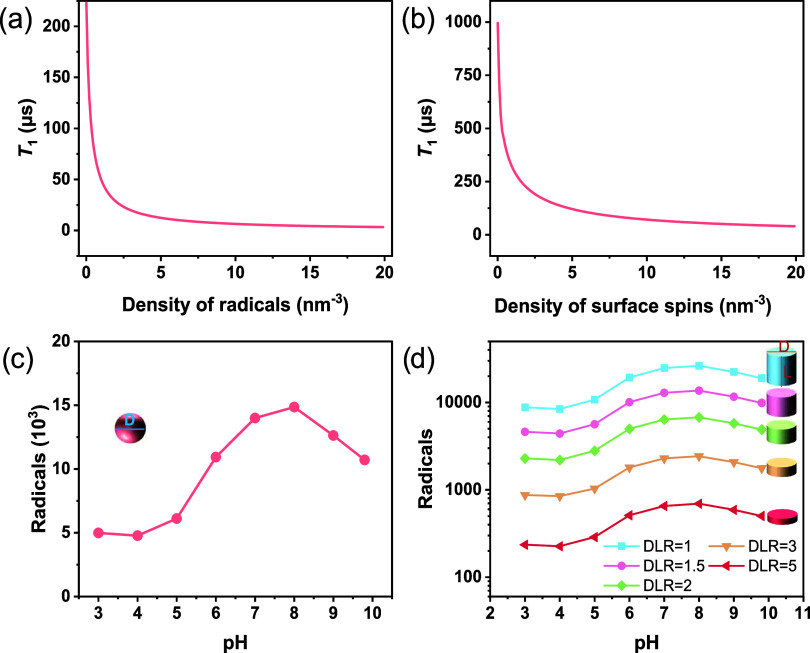
(a) Original *T*_1_ time as a function of the density of noise spins on the diamond surface, which was assumed to have a 0.1 nm thick shell (intrinsic thickness of spin on NDs). To have an average *T*_1_ of 223.9 μs for NDs with a diameter of about 27.9 nm, this density of electron spins is ρ_noise_ = 1.9/nm^3^. (b) With ρ_noise_ = 1.9/nm^3^ of the density of the noisy electron spins on the surface in (a), the *T*_1_ time as a function of the density of the electron spins in the outer 2 nm-thick coating. (c) Estimated number of radicals in the eumelanin layer for different pH values, assuming NDs are in a spherical shape. (d) Estimated number of radicals in the eumelanin layer for different pH values assuming NDs are in a disk-like shape. DLR = 2*r*_ND_/*l*_ND_. For DLR = 1.5, the volume of one disk-like ND equals to one spherical ND. The radical numbers reproduce simulated average *T*_1_ times that match the ones in Figure 2c, for samples of NDs that have a diameter of 27.9 nm and contain a NV^–^ center of random position and orientation. See Supporting Information for details of the model and simulation.

From the *T*_1_ relaxation times, we were able to estimate the number of radicals in the shell of the RGS-NDs. To achieve this, we employed a theoretical model^17,24,43^ to simulate the *T*_1_ relaxation times for different radical concentrations in the eumelanin shell. The radicals in the polymer layer produced a fluctuating magnetic field at the position of the NV^–^ center, which was characterized by an amplitude variance of *B*_⊥_^2^ and a temporal correlation time of τ_c_. Increasing concentrations and numbers of radicals produced a stronger noise amplitude, which induced a shortening of the *T*_1_ relaxation time. Let *T*_1_^other^ be the NV^–^ spin relaxation time without the radicals in the shell of a RGS-ND, the overall *T*_1_ is determined by
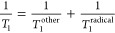
where  is the radical-induced relaxation rate of a NV^–^ center with a resonance frequency ω_NV_ ≈ 2π × 2.87 GHz. To simulate *B*_⊥_^2^ and τ_c_, we first adapted the simple spherical model, which was often used in the past years.^43,44^ We assumed that each ND has a spherical shape with a radius *r*_ND_ = 27.9/2 nm (TEM image in Figure S1a, 27.9 ± 13.1 nm, *n* = 50) and the 14 inner NV^–^ centers^45^ have random locations and orientations. We assumed that the NV^–^ centers are located at least 2 nm below the diamond surface as shallower NV^–^ centers are not stable.^46^ The eumelanin shell has a thickness of 2 nm. Due to vibrational relaxation and interradical flip–flop interactions, the magnetic field from each radical in the shell will fluctuate and contribute to the total magnetic field amplitude variance *B*_⊥_^2^ = ∑_*j*_*B*_⊥,*j*_^2^. Here, the radical and the NV^–^ have a relative distance of *r*_c,*j*_ and an orientation of *r̂*_c,*j*_. μ_0_ is the vacuum permeability, γ_e_ is the electron gyromagnetic ratio, and the unit vector *ẑ* denotes the NV^–^ symmetry axis. In our model, we considered surface electrons on the ND’s surface, which generate magnetic noise in a similar manner as the radicals in the shell of eumelanin and contribute spin relaxation in  (see Supporting Information (SI) for details of the model).
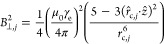
We performed Monte Carlo simulations for 1000 ND samples, where the NV^–^ center has a random location and orientation. First, we determined the density of the surface electrons to match the experimentally observed average *T*_1_^other^ of 223.9 μs. Then, we added radicals to the 2 nm shell of eumelanin on RGS-ND so that the average *T*_1_ reached the values depicted in Figure 3c. From the corresponding radical densities, we were able to estimate the number of radicals at different pH values as shown in Figure 4. The number of radicals in the eumelanin layer increased from pH 4 to 9.8, and we could estimate about 4992 (pH 3), 4777 (pH 4), 6113 (pH 5), 10,929 (pH 6), 13,992 (pH 7), 14,853 (pH 8), 12,627 (pH 9), and 10,713 (pH 9.8) radicals per spherical RGS-ND.

However, according to the TEM and AFM characterization (in Figure S1), the NDs have more disk-like shapes, which is in accordance with the most recent experimental findings of Eldemrdash et al.,^47^ who reported that the disk-like shape (or rod-like shape) could represent a better model for NDs. Therefore, we first assessed the impact of different anisotropic ND shapes with varying ratios 2*r*_ND_/*l*_ND_ of 1, 1.5, 2, and 3 on the number of radicals in the eumelanin layer based on the average *T*_1_ values depicted in Figure 3c. The obtained results are given in Table S1. Interestingly, if the volume of disk-like NDs was the same as spherical NDs (Figure 4, that is, when 2*r*_ND_/*l*_ND_ = 1.5), then the estimated detected number of radicals in the eumelanin layer is similar for different pH values. However, if the volume of the disk-like NDs became larger (smaller), which corresponds to a smaller (larger) ratio 2*r*_ND_/*l*_ND_, then the number of radicals was larger (smaller) as well. Based on our experimental data, we further simulated the NDs as disks of radius *r*_ND_ and length *l*_ND_ as depicted in Figure 4d. According to the TEM and AFM results, the average diameter of the NDs was 27.9 ± 13.1 nm (*n* = 50) and the average thickness was 5.65 ± 1.47 nm (*n* = 25) resulting in a calculated ratio 2*r*_ND_/*l*_ND_ of around 5. Therefore, we carried out the simulation based on this result. With the number of radicals in the eumelanin layer increased from pH 4 to 9.8, we could estimate about 235 (pH 3), 225 (pH 4), 287 (pH 5), 510 (pH 6), 652 (pH 7), 692 (pH 8), 589 (pH 9), and 500 (pH 9.8) radicals per RGS-ND, (Table S1). These values are much smaller than the results that were based on the spherical model due to the closer distance between the radicals and the NV^–^ centers in the disk-like model.

In general, the shapes of the curves shown in Figure 4d remained similar for all simulated plots despite the differences in the ND shapes. Although the simulated number of radicals may vary because the simulation uses approximations to account for the quantum many-body effects that would otherwise be intractable, previous results^17,24,43^ suggest that this simulation method can still provide quantitative (see Figure 1b in details) agreement between the simulated and experimental results. Furthermore, the variation of the number of radicals depends on the different ratio 2*r*_ND_/*l*_ND_. If the exact ratio 2*r*_ND_/*l*_ND_ of the NDs is not known and falls within an interval, only an interval of the radical number can be calculated based on the interval of the ratio 2*r*_ND_/*l*_ND_. This interval thus provides an estimate of the uncertainty due to the variable ND geometries in these calculations.

### Quantification of Radical Formation in the Eumelanin Shell of RGS-NDs in Living Cells

**Figure 5:**
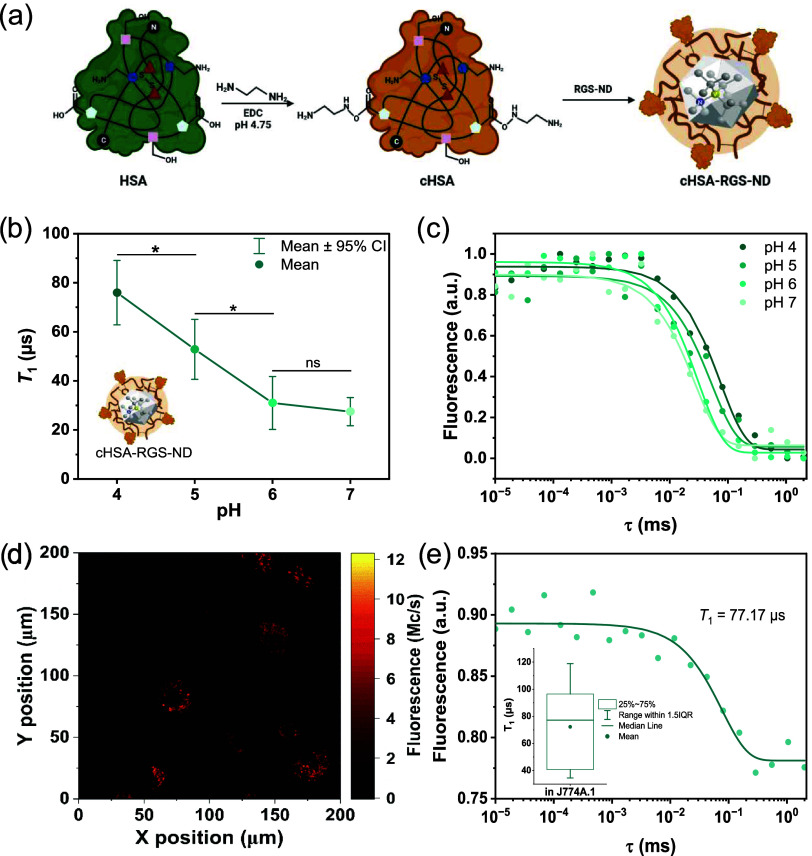
(a) Synthesis of cHSA-RGS-ND. (b) *T*_1_ relaxation time of cHSA-RGS-ND in pH 4–7 buffer solutions (*n* ≥ 15). (c) Typical *T*_1_ measurement of cHSA-RGS-ND in pH 4–7 buffer solutions. (d) *X*–*Y* axis confocal microscopy images of cHSA-RGS-ND taken up into J774A.1 cells at 100 μg/mL after 4 h incubation. (e) Representative *T*_1_ decay of cHSA-RGS-ND in macrophages, inserted is a box chart of *T*_1_ of internalized cHSA-RGS-NDs. The significance level was 0.05 for mean comparison, *p < 0.05, ***p < 0.001, ns = not significant.

Nanodiamond quantum sensing provides the unique opportunity to accurately assess the physical and chemical parameters in warm, wet and noisy environments, and even in complex systems such as living cells.^48^ Therefore, RGS-NDs were incubated with the cationic human serum albumin (cHSA), an established transporter protein that facilitates cellular uptake by clathrin-mediated endocytosis and adsorbs to NDs by forming stable complexes.^49−51^ The cHSA-RGS-NDs were prepared simply by mixing 400 μg of cHSA and 100 μL of 1 mg/mL RGD-ND for 30 min at room temperature (Figure 5a, see SI for details). After three cycles of purification by centrifugation/suspension (yield: 5.7%), the cHSA-RGS-NDs were characterized by ATR-FTIR, DLS, and ζ potential (Figure S7). The hydrodynamic diameter in water increased from 43.9 ± 1.1 to 68.0 ± 8.0 nm, while the ζ potential changed from −33.3 ± 1.7 mV for RGS-NDs to +18.3 ± 0.1 mV for the cHSA-RGS-NDs, indicating the formation of nanoparticles with positive net charges due to the adsorption of positively charged cHSA. Moreover, the cHSA-RGS-NDs were further characterized by ATR-FTIR, and the characteristic signals of the amide I and II bonds of cHSA at 1645 and 1543 cm^–1^, respectively, were clearly observed (Figure S7) indicating successful cHSA coating.

We then tested the radical generating and sensing abilities of the cHSA-RGS-ND at pH 4–7 buffer solutions over a pH range reflecting the physiological pH level in living cells. Before starting the measurement in buffer, we equilibrated the sample for more than 20 min. Like RGS-NDs, the cHSA-RGS-NDs revealed a decrease in *T*_1_ with an increasing pH level (Figures 5b,c and S8). The mean *T*_1_ relaxation times dropped from 76.0 ± 26.4 μs (pH 4), 52.8 ± 24.6 μs (pH 5), and 31.0 ± 19.5 μs (pH 6) to 27.5 ± 11.5 μs (pH 7) in buffer solutions. Based on the simulations in Figure 4, we then quantified the number of local radicals that were generated at different pH values, and 149 (pH 4), 244 (pH 5), 454 (pH 6), and 536 (pH 7) radicals were detected by the RGS-NDs, respectively. These experimental results correspond well to the data depicted in Figure 3c, also suggesting that the transporter protein cHSA did not shield the radical generating and sensing abilities of our sensor.

To study the radical formation within the eumelanin layer in living cells, cHSA-RGS-NDs were incubated with the J774A.1 macrophage cell line. After 4 h of incubation, the cells were washed three times with Dulbecco’s phosphate-buffered saline and they were maintained in colorless Leibovitz’s L-15 medium for immediate measurement of the *T*_1_ relaxation time of cHSA-RGS-NDs, which was measured on a home-built, confocal microscope as described in the SI. In brief, the *T*_1_ time was determined by first initializing the NV to the *m*_s_ = 0 state using a green laser pulse. Following a variable waiting time τ, the NV spin state was read out using a subsequent laser pulse. A mean *T*_1_ of 72.2 ± 31.0 μs was recorded (Figure 5e) corresponding to about 154 radicals formed within the eumelanin layer of intracellular RGS-NDs. TEM images of the cHSA-RGS-NDs revealed that the nanoparticles remained inside vesicles (Figure S9), which corresponds to previous data.^52^ Based on the observed number of radicals in the eumelanin layer, the intracellular pH value in the surroundings of the cHSA-RGS-NDs could be estimated. A calculated pH between 4 and 5 indicates that the cHSA-RGS-NDs were localized in lysosomal compartments with a typical pH between 4.5 and 5, compared to early (pH 6.5) or late (pH 5.5) endosomal vesicles or the cytosolic pH of 7–7.5.^53−55^ Thus, the *T*_1_ relaxation time indicated a lysosomal localization of the cHSA-RGS-NDs. Besides, the effect of UV and NIR on the *T*_1_ relaxation time was also tested (Figure S10). Briefly, the initial cellular free radical load was measured by *T*_1_ relaxometry measurements of the cHSA-RGS-ND for 15 min. Subsequently, UV (0.3 mW/cm^2^) or NIR (350 mW/cm^2^) were introduced to the cells during the continuous *T*_1_ relaxometry measurements, which were performed for an additional 10 min. For both UV and NIR, no significant differences on *T*_1_ were observed during the 10 min irradiation period, highlighting that RGS-NDs selectively respond to changes in local radical formation, and UV irradiation and local temperature fluctuations do not affect the radical generating and sensing ability of the sensor in living cells. However, very intense or extended irradiation period could in principle stimulate the formation of intracellular radicals, which, however, is beyond the scope of this study.

## Conclusions

In conclusion, we polymerized eumelanin on the surface of a nanodiamond quantum sensor. Due to the intrinsic sensitivity of the NV^–^ quantum sensor to magnetic field fluctuations, these NDs could serve as nanoscale sensors that are capable of quantitatively measuring the number of radical species in eumelanin. This was even possible at the level of individual cells, a regime inaccessible to standard EPR spectroscopy at such low radical levels. Combining the experimental *T*_1_ with theoretical simulations, we demonstrated that the number of radicals formed in the eumelanin layer is pH-dependent. Based on experimental data, we used a disk-like model with DLR = 5 to quantify the number of radicals at different pH levels from pH 3 to 9.8. The number of radicals in the eumelanin layer increased from 225 at pH 4 to 692 radicals at pH 8, respectively, and ND relaxometry was not affected by UV or NIR irradiation and light-induced heating. However, our results show that the calculated amount of radical depends very much on the geometry of the NDs. Due to the large heterogeneity of ND shapes, we therefore propose to determine the average dimensions of the NDs experimentally, as the morphology of the NDs has a significant influence on sensitivity and also affects the number of radicals detected. The availability of more uniform and regular-shaped NDs could reduce the observed deviations due to the ND geometry in the future. In comparison to EPR spectroscopy that provides valuable information on the presence and nature of paramagnetic species in various systems at the macroscopic level, we believe that *T*_1_ relaxometry could become an important tool for studying chemical reactions involving paramagnetic species in a nanoscale confined space that is not accessible by conventional techniques.

Using highly sensitive *T*_1_ relaxometry, we were able for the first time to monitor the chemical reaction in a layer of eumelanin just a few nanometers thick, even inside cells. We could quantify the number of radicals within the eumelanin shell even in a single living cell, and we estimated the local pH value based on the number of detected radicals. It is the first time that radical species were detected in eumelanin with single-cell resolution. Therefore, we believe that our method will shed light on the role of eumelanin in pigmentation, free radical scavenging, and antioxidation, which could also provide new insights into the melanin-related diseases to develop effective medical treatments.

## Supporting Information Available

Characterizations of RGS-ND and cHSA-RGS-ND; confocal image of RGS-ND in dry condition; simulation results based on different shape of NDs; *T*_1_ comparison between cHSA-RGS-ND and RGS-ND (PDF)
