# Supplementary material for: Unraveling Eumelanin Radical Formation by Nanodiamond Optical Relaxometry in a Living Cell
Source: J Am Chem Soc. 2024 Mar 12;146(11):7222–32. doi: 10.1021/jacs.3c07720 (PMC10958502; doi:10.1021/jacs.3c07720)
Supplement: Supplementary file 1 — ja3c07720_si_001.pdf [file ja3c07720_si_001.pdf]

Supporting information

Unraveling Eumelanin Radical Formation by Nanodiamond Optical Relaxometry in a Living Cell

Qi Lu [a†], Berlind Vosberg [a†], Zhenyu Wang [b,c,d], Priyadharshini Balasubramanian [e], Maabur Sow [e], Carla Volkert [a], Raul Gonzalez Brouwer [e], Ingo Lieberwirth [a], Robert Graf [a], Fedor Jelezko [e*], Martin B. Plenio [b*], Yingke Wu [a*], Tanja Weil [a*]

† Q. L and B. V contributed equally

a Max Planck Institute for Polymer Research, Ackermannweg 10, 55128 Mainz, Germany

*E-mail: weil@mpip-mainz.mpg.de, wuyingke@ mpip-mainz.mpg.de

b Institute of Theoretical Physics and Center for Integrated Quantum Science and Technology (IQST), Ulm University, Albert-Einstein-Allee 11, 89081 Ulm, Germany

*E-mail: martin.plenio@uni-ulm.de

c Key Laboratory of Atomic and Subatomic Structure and Quantum Control (Ministry of Education), and School of Physics, South China Normal University, Guangzhou 510006, China

d Guangdong Provincial Key Laboratory of Quantum Engineering and Quantum Materials, and Guangdong-Hong Kong Joint Laboratory of Quantum Matter, South China Normal University, Guangzhou 510006, China

e Institute for Quantum Optics and Center for Integrated Quantum Science and Technology (IQST), Ulm University, Albert-Einstein-Allee 11, 89081 Ulm, Germany

*E-mail: fedor.jelezko@uni-ulm.de

Contents

1. Experimental section 3
1.1. Materials 3
1.2. Methods 3
1.2.1. Preparation of RGS-ND 3
1.2.2. Preparation of cHSA-RGS-ND 3
1.2.3. Transmission Electron Microscopy (TEM) 4
1.2.4. Dynamic Light Scattering (DLS) and Zeta potential 4
1.2.5. Attenuated total reflection-fourier transform infrared (ATR-FTIR) spectroscopy 4
1.2.6. UV-Vis spectroscopy 4
1.2.7. Atomic force microscopy 4
1.2.8. Electron spin resonance (ESR) spectroscopy 5
1.2.9. T_1 relaxation time measurement 5
1.2.10. Cell culture 7
1.2.11. T_1 relaxation time measurement in living cell 7
1.2.12. Simulation of spin relaxation times 8
1.2.13. Samples preparation of cell with cHSA-RGS-ND for Transmission Electron Microscopy (TEM) 9
1.2.14. Statistics analysis 10
2. Figures and tables 11
3. References 20

1. **Experimental section**

1.1. Materials

Nanodiamonds were purchased from Adámas Nanotechnologies; L-DOPA and $NaIO_4$ were purchased from Sigma-Aldrich, H_2O was obtained from the Millipore purification system. Dulbecco's Modified Eagle's Medium (DMEM, 1x), Leibovitz's L-15 Medium, Dulbecco's phosphate-buffered saline (DPBS, 1x), fetal bovine serum (FBS), and Penicillin Streptomycin (Pen Strep) were purchased from Gibco. A549 cells from human adenocarcinoma lung tissue and murine monocytes-macrophages cell line J774A.1 were purchased from the German Collection of Microorganisms and Cell Cultures GmbH (DSMZ). All solvents and chemicals were purchased from commercial sources and were used without further purification.

1.2. Methods

1.2.1. Preparation of RGS-ND

RGS-NDs were prepared according to our previous report with slight adjustments.[1] Briefly, 100 µL 1 mg/mL ND water dispersion from Adámas Nanotechnologies was diluted with 811 µL MilliQ water and mixed with 79 µL 2.5 mg/mL L-DOPA. After 3 minutes sonication, 10 µL 10.84 mg/mL $NaIO_4$ was added, followed by 15 minutes shaking. The resulting RGS-ND was purified by 3 cycles of centrifugation/suspension.

1.2.2. Preparation of cHSA-RGS-ND

Cationic human serum albumin was prepared according to our previous work.[2-4] Briefly, 150 mg of HSA was dissolved completely in 15 mL of degassed ethylenediamine-HCl solution (2.5 M, pH 4.75), EDC (4 mmol, 621 mg) was then added and stirred for 2 hours. The reaction was terminated by adding an acetate buffer (1 mL, 4 M, pH 4.75). After reaction, the cHSA was washed twice with acetate buffer (4 M, pH 4.75) and 3 times with deionized distilled water using Vivaspin 20 (30 kDa MWCO) centrifugal concentrator and then lyophilized to obtain cHSA as a white fluffy solid. The cHSA-RGS-NDs were prepared simply by mixing 400 µg cHSA and 100 µL

1 mg/mL RGD-ND for 30 minutes at room temperature, and purified by 3 cycles of centrifugation/suspension.

1.2.3. Transmission Electron Microscopy (TEM)

One drop of a 0.1 mg/mL solution of sample in MilliQ was placed onto an oxygen treated copper grid and dried at room temperature. A Jeol 1400 transmissions electron microscope was used to obtain bright field images

1.2.4. Dynamic Light Scattering (DLS) and Zeta potential

500 µL of 0.1 mg/mL solutions of RGS-ND in MilliQ were transferred into a borosilicate glass cuvette and measured at 25 °C with a 90° angle using a particle sizer Zetasizer Nano Z (Malvern Panalytical). The hydrodynamic diameter distribution was presented as intensity. The Zeta potential was measured in water at 25 °C with a Zetasizer Nano Z (Malvern Panalytical).

1.2.5. Attenuated total reflection-fourier transform infrared (ATR-FTIR) spectroscopy

10 µL of 1 mg/mL solutions of RGS-ND in MilliQ were dropped on aluminum foil and dried overnight. The IR spectrum was measured from 4000 to 400 cm^{-1} with a resolution of 4 cm^{-1} in absorbance mode on a FT-IR Spectrometer TENSOR II equipped with Platinum ATR (Bruker).

1.2.6. UV-Vis spectroscopy

1 µL of 0.1 mg/mL solutions of RGS-ND in MilliQ were subjected to NanoDrop™ 2000/2000c Spectrophotometers (Thermo Fisher Scientific). The absorbance was recorded from 200 to 800 nm with 1 nm resolution.

1.2.7. Atomic force microscopy (AFM)

Atomic force microscopy was conducted in air with a Bruker Dimension Icon atomic force microscope, which was operated in Tapping mode. AFM probes with a nominal spring constant of 26 N/m, corresponding to a resonance frequency of 300 kHz (OTESPA, Bruker) were used. The

samples were diluted with MilliQ water to a concentration of 0.05 mg/mL. Sample solution (30 µL) was added onto a freshly cleaved and plasma cleaned mica substrate (circular, 15 mm) and incubated for at least 10 min to allow deposition of the structures. Remaining solution was removed. Samples were scanned with scan rate 300 kHz and scan sizes between 0.5 and 5 µm. Images were processed with the NanoScope Analysis software (Version 1.9).

1.2.8. Electron spin resonance (ESR) spectroscopy

CW (continuous wave) X-Band (~ 9.4 GHz) ESR spectra were recorded using a MiniScope MS200 from Magnettech. The frequency stability of the spectrometer was controlled with a frequency counter from Hewlett Packard. All cw ESR measurements were performed at ambient conditions with 50 mW micro wave power, a field modulation amplitude of 400 µT and a modulation frequency of 100 kHz. A scanning duration of 600 s/scan was chosen and 16 transients were accumulated for each spectrum. The pH dependent RGS-ND samples with concentration of 1 mg/mL were prepared dispersing RGS-NDs in pH buffer solutions. For the measurements 20 µL sample solution is filled in a 50 µL micropipette capillary from BLAUBRAND and sealed using Critoseal. As the NDs are not really dissolved in the solution, they sediment in these tubes during measurements. Therefore, it is important to place the sedimentation layer of the RGS-NDs reproducibly in the center of the micro wave cavity of the ESR spectrometer. For the processing of the recorded data, the EasySpin[5] package for Matlab and Origin Software was employed.

1.2.9. T_1 relaxation time measurement

The longitudinal spin relaxation time (T_1) of the NV centers in the nanodiamonds was measured using a home-built confocal fluorescence microscope. The NV centers were excited using a 532 nm laser, which was focused onto the sample using an oil-immersion objective (Nikon PLAN

100x oil, N.A. = 1.35). The resulting fluorescence from the NV centers was collected by the same objective and filtered with a 740/75 nm band-pass filter and detected using an avalanche photodiode (APD).

The pulse sequence for the T_1 time measurement is shown in Figure 3a. It consisted of a series of 10 μs long laser pulses. The laser pulse polarizes the NV centers in the $m_s = 0$ spin state. After a variable waiting time (τ), the subsequent laser pulse reads out the spin state of the NV centers. The fluorescence photons detected in the first 300–500 ns of the laser pulse contained the spin state information and hence constitute the signal. The T_1 measurement data shown were normalized, i.e., the signal (fluorescence obtained during the first 300 ns) was divided by the reference steady-state fluorescence (fluorescence obtained when the NV center was re-initialized into the $m_s = 0$ spin state). The T_1 measurement sequence was repeated several times with a total acquisition time of 15 minutes. The measured fluorescence data are plotted as a function of the waiting time (τ) between the laser pulses and fitted with a mono-exponential function of the form $I_0 + A \exp{(\tau/T_1)}$.

For the T_1 time statistics, the ND samples were prepared by placing 10 μL of 0.01 mg/mL^{-1} samples in the silicone gasket placed on top of an O2-plasma cleaned glass coverslip. The samples were dried overnight and T_1 measurements were performed using the home-built confocal microscope described above (See Figure 1, Created with BioRender.com). For the T_1 measurements, only single particles with a moderate count rate (500,000 ± 300,000) were measured. 10 mM different pH buffer solutions (pH 3: citrate, pH 4 and 5: acetate, pH 6-9.8: phosphate) were added when needed. A total of 20 single, isolated fluorescence spots were selected for the T_1 measurement and some data points are excluded based on the relevance of fit (R-squared < 0.9).

1.2.10. Cell culture

A549 and J774A.1 cells were cultured in DMEM supplemented with 10% FBS and 1% Penicillin/Streptomycin in a humidified atmosphere at 37 °C and 5% CO_2. Fresh culture medium was replaced every two days and cells were sub-cultured after reaching 80% confluence.

1.2.11. T_1 relaxation time measurement in living cell

J774A.1 cells were seeded in an ibidi 18 well µ-slide (100,000 cells/mL, 100 µL each well). After overnight incubation, cells were washed with DPBS, then 100 µg/mL cHSA-RGS-ND in culture medium were added to the cells (100 µL in each well). Then cells were incubated at 37 °C again for 4 hours, washed 3 times with DPBS and maintained in colorless Leibovitz's L-15 Medium for immediate T_1 relaxation time measurements. T_1 measurements were performed on a home-built confocal microscope as described in a previous report[6] but with the following differences. A 40X oil-immersion objective with a 1.4 NA from *Olympus* (UPLXAPO40XO) was used. Laser pulses for T_1 measurements were created with an arbitrary waveform generator 70001A from *Tektronix* and a directly modulated 513 nm laser from *Toptica Photonics* (Ibeam-smart 515.S-15133). The laser beam was filtered by a 513 nm bandpass filter (HQ515/20M, *Chroma*), then a lambda half plate (AHWP10M-600, *Thorlabs*) followed by a polarizing beamsplitter (PBS121, *Thorlabs*) were used to further control the laser power. A piezo stage (P-562.3CD) from *Physik Instrumente* was implemented for the objective's positioning. Fluorescence pulses were collected by an *Excelitas* avalanche photodiode (SPCM-AQRH 13) protected by a 590 nm long pass filter (ET590lp, *Chroma*). Pulse averaging was executed with a *FAST ComTec* time tagger (MCS6A1T2) and a *National Instruments* card (6343) allowed us to manage the analogue/digital interfacing between the computer and the microscope. To control the temperature, an objective heater (Objektivheizer 2000) and heated insert (P Lab-TekTM S1) from *Pecon* was implemented. The entire experiment was controlled by the customized open source software: Qudi[7]. The single particle-tracking algorithm used was inspired from the works of Feng et al.[8]

For the UV and NIR irradiation, the initial intracellular free radical load was measured by T1 relaxometry measurements on the cHSA-RGS-ND for 15 min. Subsequently, UV (0.3 mW/cm^2) or NIR (350 mW/cm^2) were introduced to the cells during the continuous T1 relaxometry measurements, which were performed for extra 10 minutes.

1.2.12. Simulation of spin relaxation times

The model[9-11] used to simulate the spin relaxation times is similar to those of previous publications. In this model, we considered a thin layer of surface electrons on the diamond surface and a thicker layer (thickness of 2 nm) of radicals on top of the diamond surface for the RGS-ND. The NDs are assumed to have a spherical shape or disk-like shape as this provides a good approximation for the simulation according to previous studies.[9-11] Both layers reduce the relaxation times of the NV centers due to fluctuations of their electron spins. Without the radicals in the shell of RGS-ND, the relaxation time T_1^{other} is given by

$$\frac{1}{T_1^{\mathrm{other}}} = \frac{1}{T_1^{\mathrm{i}}} + \frac{1}{T_1^{\mathrm{noise}}},$$

where $T_1^{\mathrm{i}} = 1$ ms is the intrinsic NV relaxation time (with a value similar to that of NVs in bulk diamond). Here the contribution $\frac{1}{T_1^{\mathrm{noise}}} = 3\gamma_e^2 {B'}_{\perp}^2 \frac{\tau_c'}{1 + (\omega_{\mathrm{NV}}\tau_c')^2}$ due to the noise originating from the surface electrons takes the same functional form as that from the radicals given in the main text. The expression for ${B'}_{\perp}^2$ (τ_c') takes the same form as $B_{\perp}^2$ (τ_c) for the radicals. Following references[9-11], the correlation time has two contributions, the first originating from flip-flop interactions between electron spins and a second from vibrational relaxation and is given by

$$\tau_c = \left(\frac{\mu_0 \gamma_e^2 (8\pi\rho)^{1/2}}{16\pi \quad r_{\mathrm{min}}^{3/2}} + R_{\mathrm{vib}} \right)^{-1},$$

where ρ is the volume density of the radicals, $R_{\mathrm{vib}} = 50$ GHz is the intrinsic vibrational spin relaxation, and $r_{\mathrm{min}} = 0.2$ nm is a parameter

describing the minimum allowed distance among the radicals. In the case of surface electron spin, we chose $r_{\min} = 0.15$ nm.

In the Monte Carlo simulation, we randomly choose NDs where the random locations and orientations of the NV centers follow uniform distributions with an exclusion range of 2nm from the diamond surface. For NDs with a diameter of 27.9 nm, a density 11 nm^{-3} of the surface electrons in a 0.1 nm thick diamond surface was used to reproduce $T_1^0 = 223.9\ \mu$s. This surface electron density was used in the simulation for the radical numbers at different pH values. Using the T_1 times in Fig. 2c of the main text, we determined the number of radicals in the shell of RGS-ND.

1.2.13. Samples preparation of cell with cHSA-RGS-ND for Transmission Electron Microscopy (TEM)

The TEM samples were prepared inline with our previous report.[6] Briefly, A549 cells were cultured in a 24-well plate pre-placed with carbon coated sapphire discs (d:3mm) with a density of 50,000 cells/mL. After 4 hours co-incubation with cHSA-RGS-ND, sapphire discs were placed between 2 aluminum plates to create a 'sandwich' and were mounted afterwards into a holder (Engineering Office, M. Wohlwend) and immediately fixated in a Wohlwend HPF Compact 01 high-pressure freezer (Engineering Office, M. Wohlwend) with a pressure of 2100 bar. The frozen samples were then stored in liquid nitrogen. Frozen sapphire discs were carefully removed from the aluminum 'sandwich' and transferred into 1 mL pre-cooled freeze substitution medium (0.2% (w/v) osmium tetroxide, 0.1% (w/v) uranyl acetate, 5% (v/v) distilled water in acetone) and kept in a freeze substitution unit (AFS2,Leica). Samples were then slowly warmed up to 0 °C over a period of 20 hours in the unit. After being warmed up, the freeze-substituted samples were increased to room temperature, then the substitution medium was removed and the discs were washed 3 times with acetone at half an hour intervals. Then the discs were infiltrated sequentially in gradient epoxy resin-acetone mixture (1:1, 1:2, and 2:1) for 1 h. Samples were then infiltrated in 100% epoxy resin overnight. Finally, each sample was

transferred into a new Eppendorf tube containing fresh epoxy resin for polymerization at 60 °C for 24 h. Following polymerization, sapphire discs were detached using liquid nitrogen. Resin blocks with imprinted cells were trimmed and partitioned into 100nm/80nm sections using a 45° diamond knife (Diatome) in EM UC6 ultramicrotome (Leica).

1.2.14. Statistics analysis

Where given, the data are presented as mean ± SD. Sample sizes are given in the Figure caption. OriginPro 2023 software from OriginLab was used for one-way ANOVA analysis, Tukey's HSD (Honestly Significant Difference) *post hoc* test, and Levene's test for equal variance. Significance level was 0.05 for mean comparison, * $p < 0.05$, *** $p < 0.001$, ns = Not significant.

2. Figures and tables

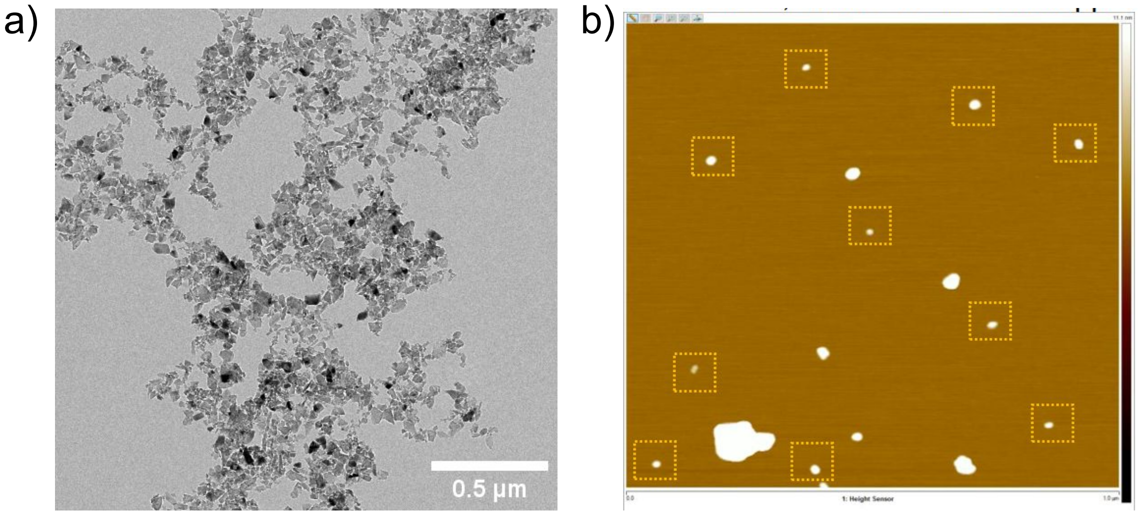

Figure S1. *a) TEM image and b) AFM image (Topography, Scale bar 11.1 nm, Roughness 1.6 nm, the height of NDs in dashed rectangles are analyzed) of fluorescent nanodiamond purchased from Adámas Nanotechnologies.*

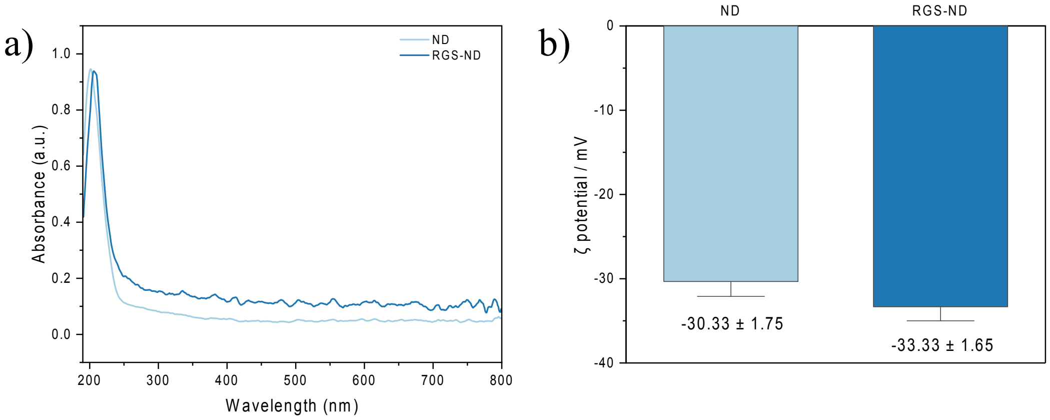

Figure S2. *Characterizations of RGS-ND. a) UV-Vis spectrum. b) Zeta potential.*

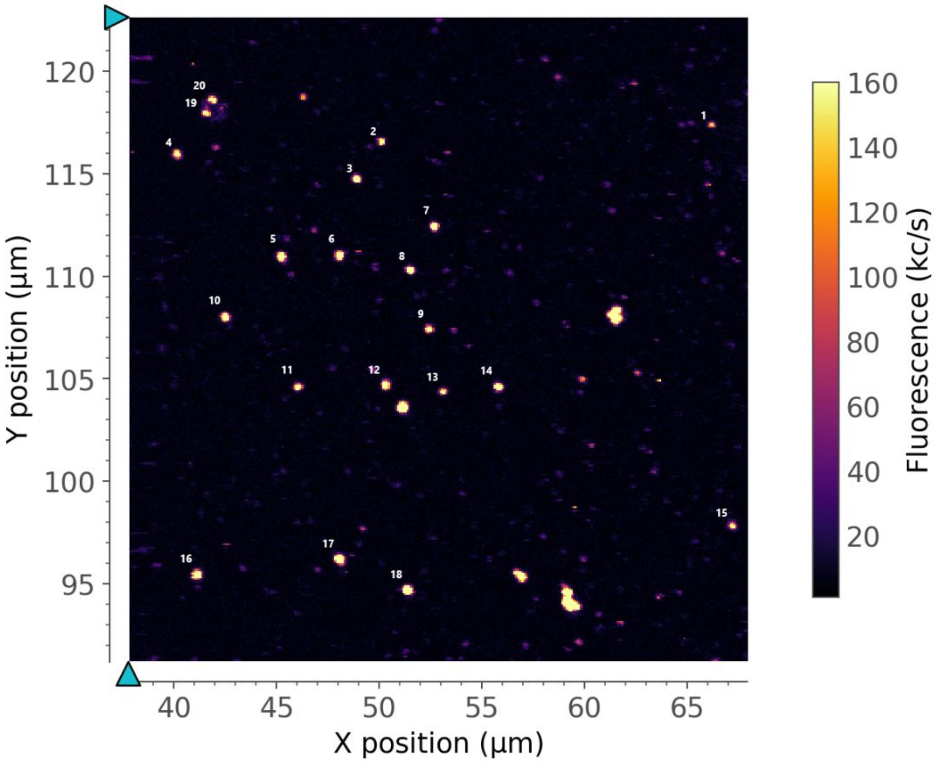

Figure S3. *Confocal image of RGS-ND in dry condition.*

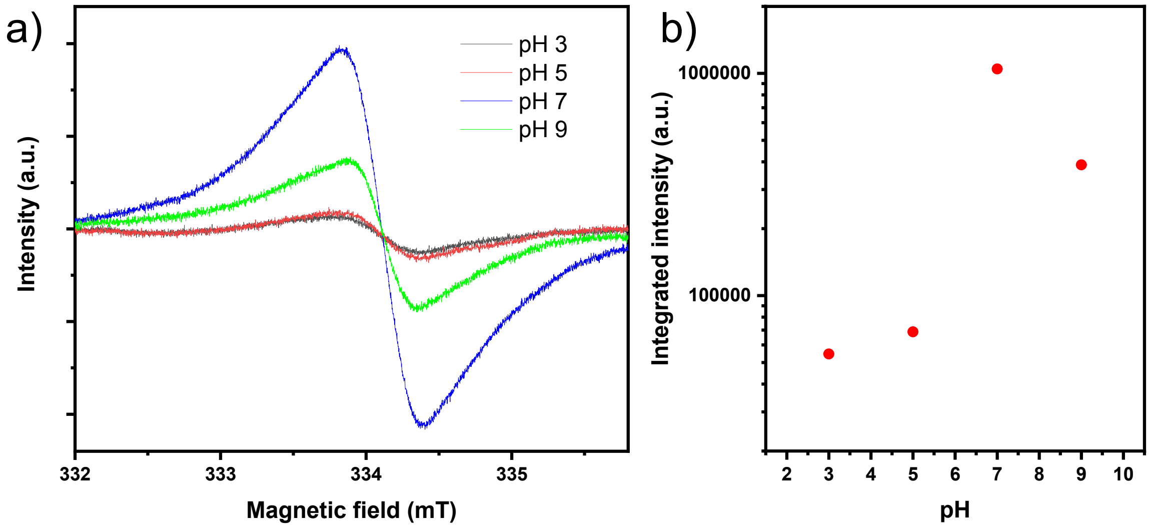

Figure S4. *a) Electron Paramagnetic Resonance spectra of RGS-ND in pH (3, 5, 7, and 9) buffer solutions. b) Integrated intensity of RGS-ND in pH (3, 5, 7, and 9) buffer solutions in a).*

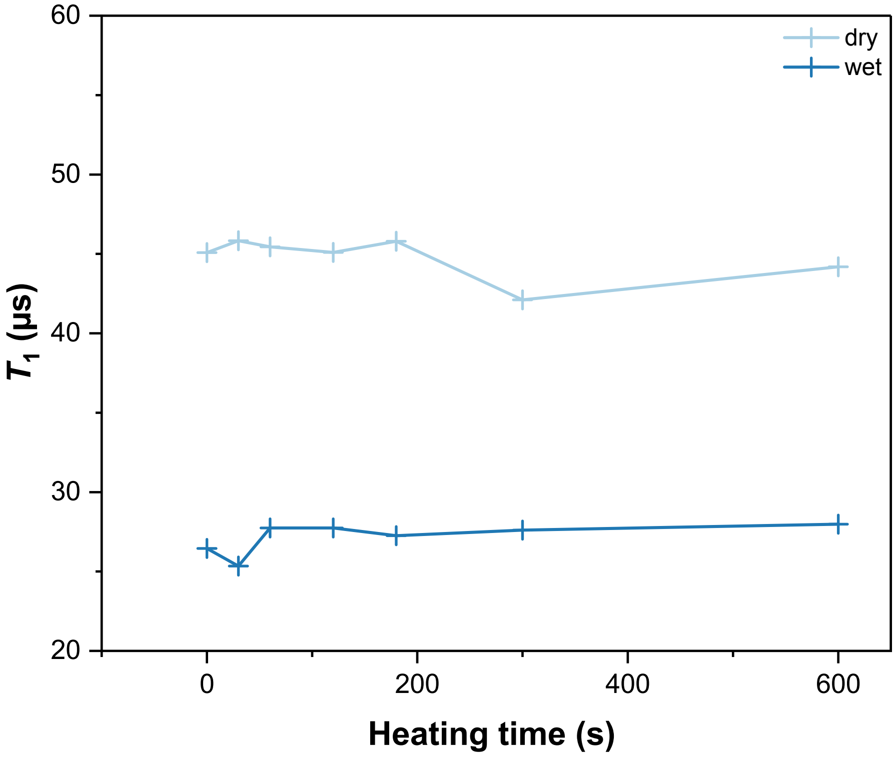

Figure S5. *T_1 of RGS-ND after NIR (810 nm) irradiation under dry and wet (in water) condition.*

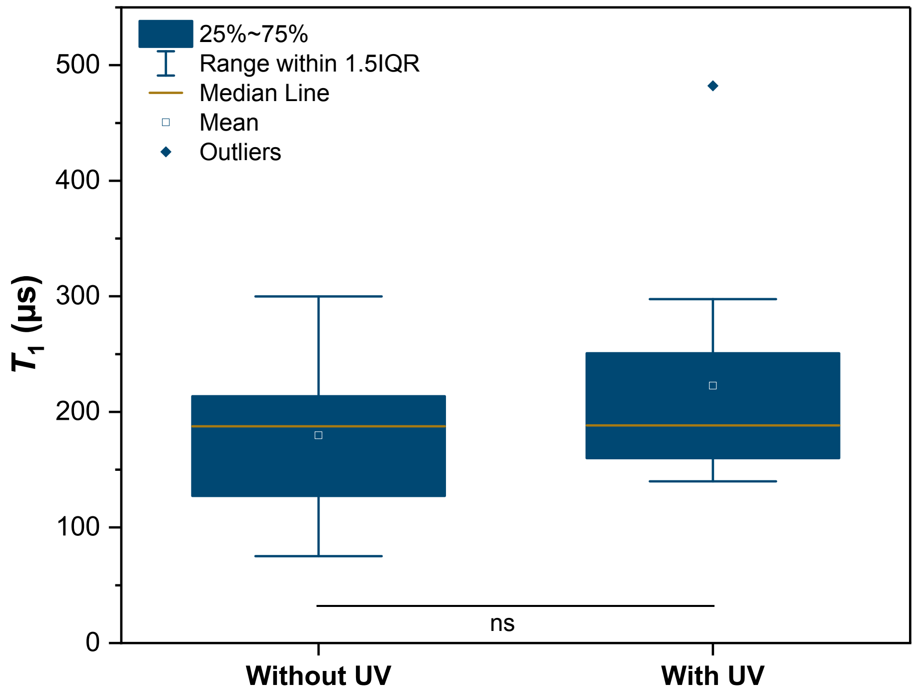

Figure S6. *T_1 of RGS-ND with or without UV (365 nm) irradiation. n≥ 13.*

a)

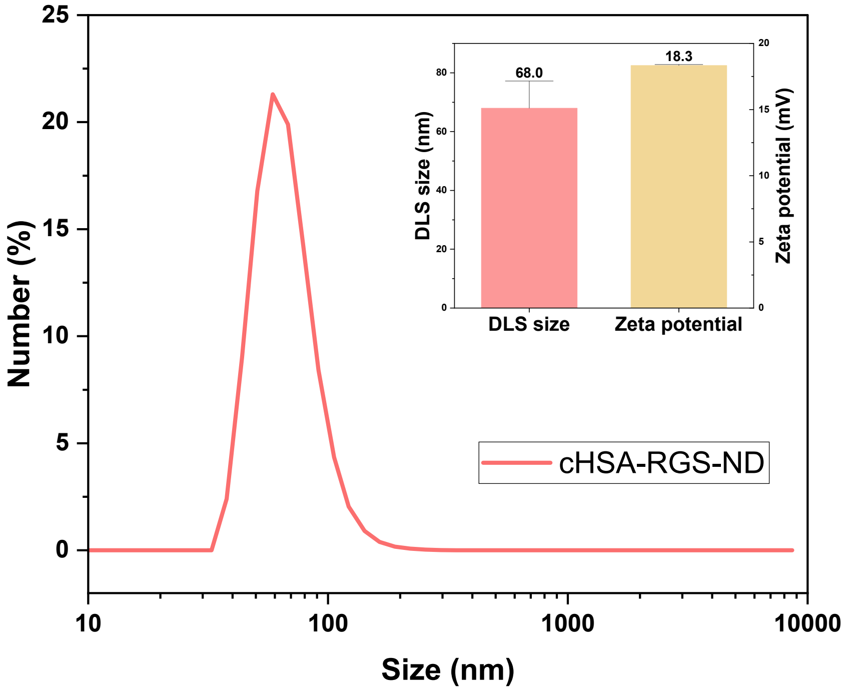

b)

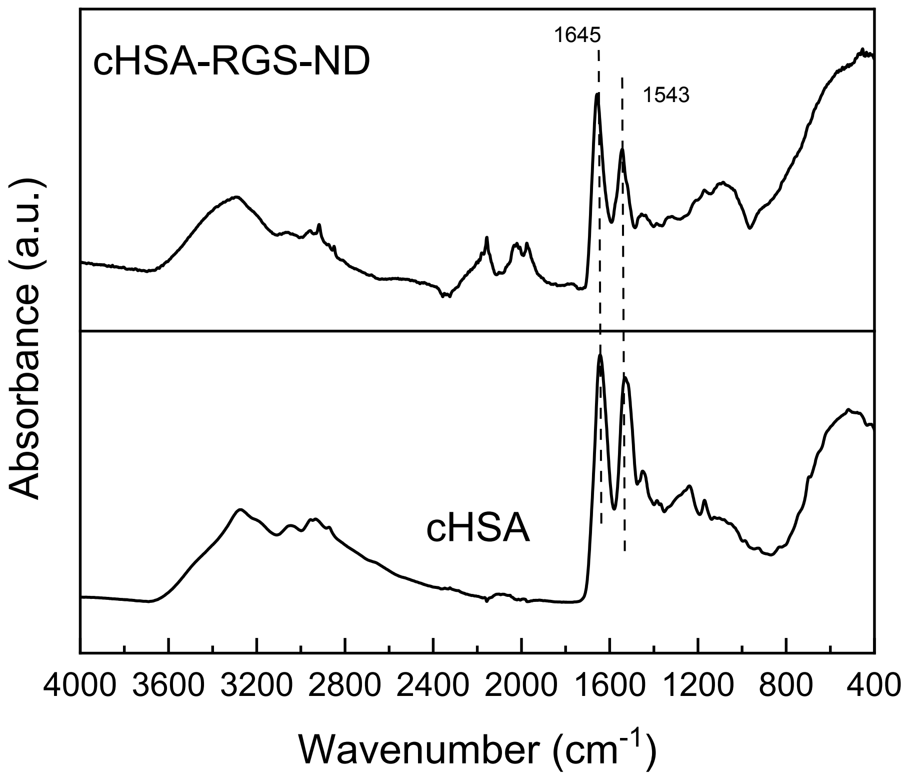

Figure S7. *Characterization of cHSA-RGS-ND. a) Hydrodynamic size distribution of cHSA-RGS-ND, average hydrodynamic size and zeta potential are inserted. b) ATR-FTIR of cHSA-RGS-ND and cHSA.*

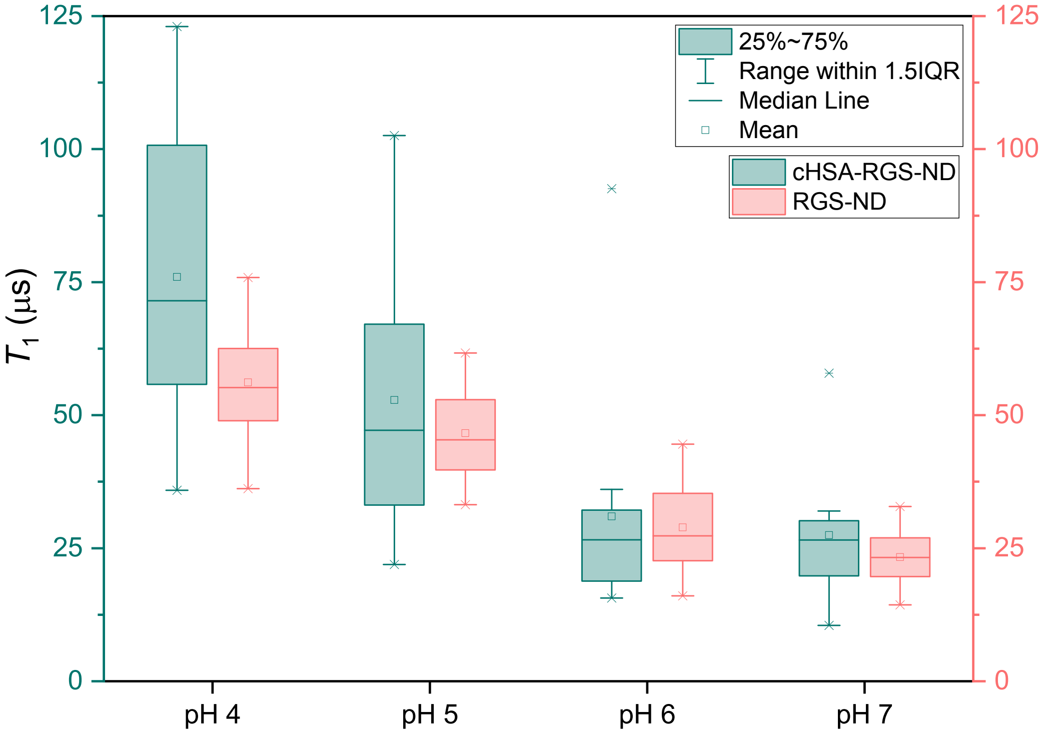

Figure S8. *T_1 comparison between cHSA-RGS-ND and RGS-ND.*

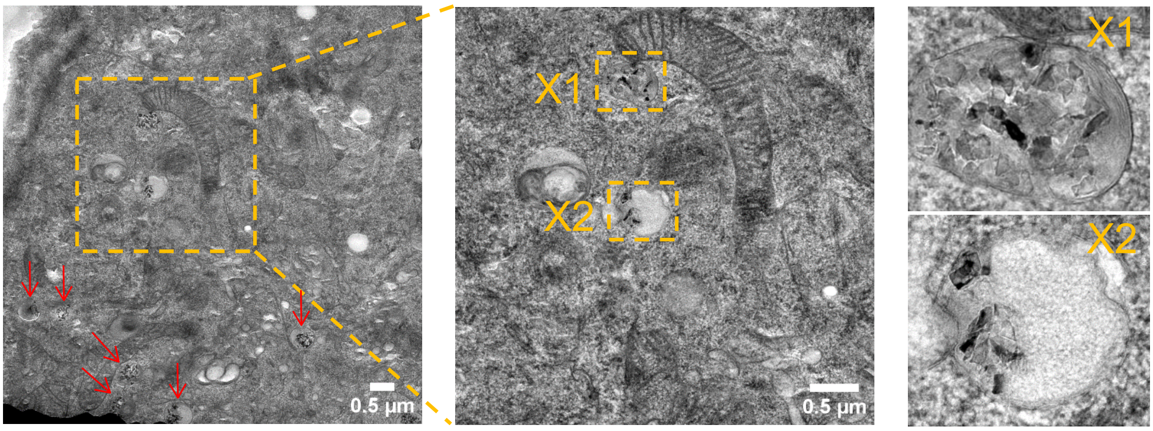

Figure S9. *TEM images of internalized cHSA-RGS-ND in A549 cells after a 4-hour incubation. cHSA-RGS-NDs are indicated with red arrows.*

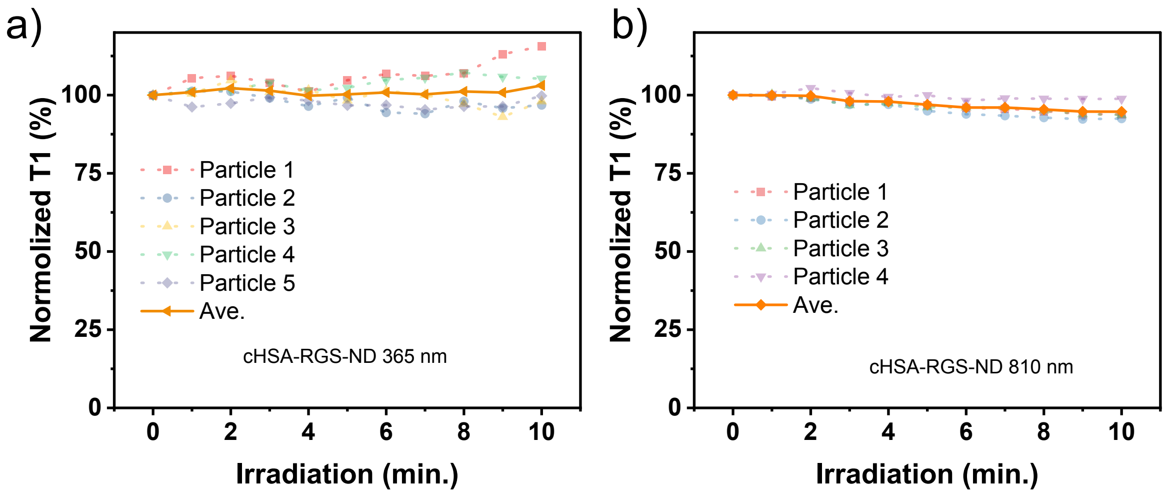

Figure S10. *Normalized T_1 changes of cHSA-RGS-ND in J774A.1 cells with UV (a. 365 nm, 0.3 mW/cm^2) and NIR (b. 810 nm, mW/cm^2) irradiation. The irradiation started after 15 minutes of initial T_1 measurement, and set as 0 minute in a) and b). The T_1 was normalized to the T_1 value at 0 minute of each particle.*

Table S1. *Simulation results based on different shape of NDs*

pH	Number of radicals					
	Spherical ND	Disk-like shape ND				
		DLR=1	DLR=1.5	DLR=2	DLR=3	**DLR=5**
	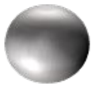	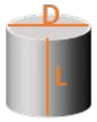	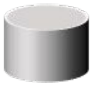	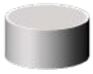	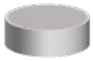	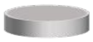
3	4992	8788	4606	2289	871	**235**
4	4778	8409	4408	2193	846	**225**
5	6114	10774	5638	2799	1031	**287**
6	10929	19313	10067	4982	1799	**510**
7	13993	24749	12882	6369	2286	**652**
8	14853	26275	13672	6758	2421	**692**
9	12627	22326	11627	5752	2069	**589**
9.8	10713	18929	9868	4884	1765	**500**

3. References

(1) Harvey, S.; Raabe, M.; Ermakova, A.; Wu, Y.; Zapata, T.; Chen, C.; Lu, H.; Jelezko, F.; Ng, D. Y.; Weil, T. Transferrin-Coated Nanodiamond–Drug Conjugates for Milliwatt Photothermal Applications. *Adv. Therap.* **2019**, *2* (11), 1900067. DOI: 10.1002/adtp.201900067.
(2) Wu, Y.; Ermakova, A.; Liu, W.; Pramanik, G.; Vu, T. M.; Kurz, A.; McGuinness, L.; Naydenov, B.; Hafner, S.; Reuter, R.; et al. Programmable Biopolymers for Advancing Biomedical Applications of Fluorescent Nanodiamonds. *Adv. Funct. Mater.* **2015**, *25* (42), 6576-6585. DOI: 10.1002/adfm.201502704.
(3) Wu, Y.; Ihme, S.; Feuring-Buske, M.; Kuan, S. L.; Eisele, K.; Lamla, M.; Wang, Y.; Buske, C.; Weil, T. A Core–Shell Albumin Copolymer Nanotransporter for High Capacity Loading and Two-Step Release of Doxorubicin with Enhanced Anti-Leukemia Activity. *Adv. Healthc. Mater.* **2013**, *2* (6), 884-894. DOI: 10.1002/adhm.201200296.
(4) Eisele, K.; Gropeanu, R. A.; Zehendner, C. M.; Rouhanipour, A.; Ramanathan, A.; Mihov, G.; Koynov, K.; Kuhlmann, C. R. W.; Vasudevan, S. G.; Luhmann, H. J.; Weil, T. Fine-tuning DNA/albumin polyelectrolyte interactions to produce the efficient transfection agent cBSA-147. *Biomater.* **2010**, *31* (33), 8789-8801. DOI: 10.1016/j.biomaterials.2010.07.088.
(5) Stoll, S.; Schweiger, A. EasySpin, a comprehensive software package for spectral simulation and analysis in EPR. *J. Magn. Reson.* **2006**, *178* (1), 42-55. DOI: 10.1016/j.jmr.2005.08.013.
(6) Wu, Y.; Alam, M. N. A.; Balasubramanian, P.; Ermakova, A.; Fischer, S.; Barth, H.; Wagner, M.; Raabe, M.; Jelezko, F.; Weil, T. Nanodiamond Theranostic for Light-Controlled Intracellular Heating and Nanoscale Temperature Sensing. *Nano Lett* **2021**, *21* (9), 3780-3788. DOI: 10.1021/acs.nanolett.1c00043.
(7) Binder, J. M.; Stark, A.; Tomek, N.; Scheuer, J.; Frank, F.; Jahnke, K. D.; Müller, C.; Schmitt, S.; Metsch, M. H.; Unden, T.; et al. Qudi: A modular python suite for experiment control and data processing. *SoftwareX* **2017**, *6*, 85-90. DOI: 10.1016/j.softx.2017.02.001.
(8) Feng, X.; Leong, W.-H.; Xia, K.; Liu, C.-F.; Liu, G.-Q.; Rendler, T.; Wrachtrup, J.; Liu, R.-B.; Li, Q. Association of Nanodiamond Rotation Dynamics with Cell Activities by Translation-Rotation Tracking. *Nano Letters* **2021**, *21* (8), 3393-3400. DOI: 10.1021/acs.nanolett.0c04864.
(9) Wu, Y.; Balasubramanian, P.; Wang, Z.; Coelho, J. A. S.; Prslja, M.; Siebert, R.; Plenio, M. B.; Jelezko, F.; Weil, T. Detection of Few Hydrogen Peroxide Molecules Using Self-Reporting Fluorescent Nanodiamond Quantum Sensors. *J. Am. Chem. Soc.* **2022**, *144* (28), 12642–12651. DOI: 10.1021/jacs.2c01065.
(10) Barton, J.; Gulka, M.; Tarabek, J.; Mindarava, Y.; Wang, Z.; Schimer, J.; Raabova, H.; Bednar, J.; Plenio, M. B.; Jelezko, F.; et al. Nanoscale Dynamic Readout of a Chemical Redox Process Using Radicals Coupled with Nitrogen-Vacancy Centers in Nanodiamonds. *ACS Nano* **2020**, *14* (10), 12938-12950. DOI: 10.1021/acsnano.0c04010.
(11) Tetienne, J.-P.; Hingant, T.; Rondin, L.; Cavaillès, A.; Mayer, L.; Dantelle, G.; Gacoin, T.; Wrachtrup, J.; Roch, J.-F.; Jacques, V. Spin relaxometry of single nitrogen-vacancy defects in diamond nanocrystals for magnetic noise sensing. *Phys. Rev. B* **2013**, *87* (23), 235436. DOI: 10.1103/PhysRevB.87.235436.
